# A Pair of Crescent-shaped Proteins Shape Vesicles at the Golgi

**DOI:** 10.1371/journal.pbio.1001543

**Published:** 2013-04-23

**Authors:** Richard Robinson

**Affiliations:** Freelance Science Writer, Sherborn, Massachusetts, United States of America

## Abstract

Two membrane proteins containing a BAR domain, PICK1 and ICA69, regulate biogenesis and maturation of insulin granules in flies and mice, and are essential for normal growth and metabolic homeostasis.

The Golgi network may look like a stack of pancakes, but it functions like a high-speed, just-in-time cargo facility, modifying, sorting, and packaging proteins for destinations within and beyond the cell. Among these proteins are hormones, such as insulin and growth hormone, that are shuttled into secretory vesicles, where they remain until the cell receives the call to ship them out. The molecular dynamics of those secretory vesicles are becoming better known, thanks to two new studies that identify a pair of interacting proteins–PICK1 and ICA69—as critical players in the formation and maturation of secretory vesicles, with potential roles in endocrine diseases.

**Figure:**
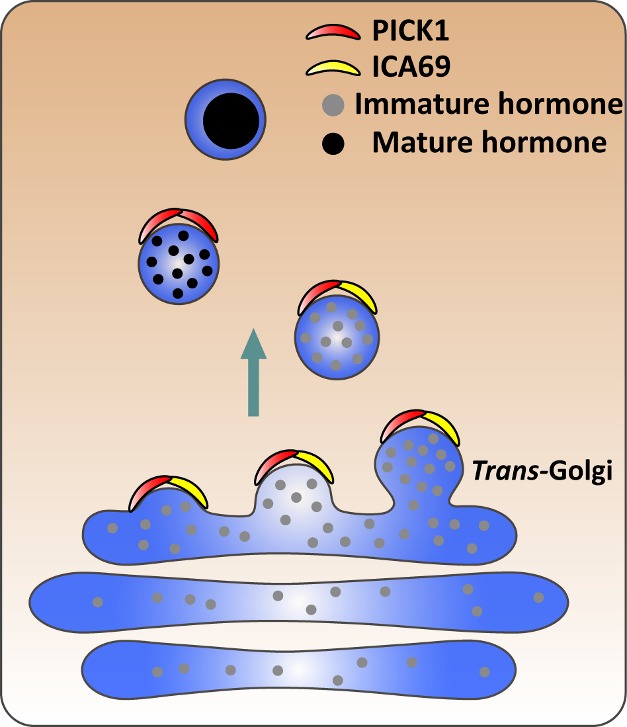
PICK1 and ICA69 from a compound structure, likely via domains that help impose curvature on membranes, to facilitate the budding and maturation of dense core vesicles from the *trans*-Golgi network.

PICK1 is known to be involved in the trafficking of membrane proteins, and has two domains that suit it for a role in vesicle formation: one that binds to membrane proteins, and another, the crescent-shaped BAR domain, which helps impose curvature on membranes. Recently, Mian Cao, Jun Xia, and colleagues reported that PICK1 binds to ICA69, another membrane protein with a BAR domain. In their new study, Cao, Xia, and Zhuo Mao explored the role of this pair in pancreatic beta cells that release insulin.

They began by showing that loss of PICK1 led to impaired glucose tolerance and lowered serum levels of insulin. Studying insulin-producing pancreatic beta cells in mice lacking PICK1, they found that while the level of insulin was low, the level of proinsulin was high. Proinsulin is packaged into vesicles in the *trans*-Golgi network, the site of secretory vesicle budding, before it is cleaved into functional insulin later on. Islet cells contained an excess of immature secretory vesicles, and a deficiency of mature ones, indicating that loss of PICK1 was interfering with the maturation of insulin-containing vesicles, accounting for reduction in serum insulin and impaired glucose tolerance. Importantly, they found the same traits, both on a cellular and organismic level, when they knocked out ICA69, arguing that the two binding partners cooperate in the maturation of insulin-containing vesicles. They also showed that as vesicles mature, they lose ICA69 while retaining PICK1.

In the second study, Birgitte Holst, Kenneth Madsen, Ole Kjaerulff, Ulrik Gether, and colleagues looked at PICK1's role in the trafficking of growth hormone from the pituitary. They found that loss of PICK1 led to growth retardation in both fruit flies and mice, and that stimulation-induced growth hormone release was deficient in mice (echoing Cao et al., they also observed impaired glucose tolerance and reduced serum insulin). Using a variety of staining techniques, they showed that PICK1 localized to immature growth hormone-containing vesicles leaving the *trans*-Golgi network. As with insulin, loss of PICK1 reduced the number of mature vesicles containing growth hormone. Mutation of PICK1's BAR domain reduced its ability to remain localized to the Golgi. In vitro, the isolated PICK1 BAR domain triggered the formation of networked tubular membrane structures, consistent with its membrane-curving function.

Holst et al. also found that ICA69 and PICK1 colocalized, and they showed that knocking down ICA69 reduced PICK1 staining in pituitary cells. Even in non-endocrine cells, expression of the two proteins promoted vesicle formation, and the pair concentrated near the outbound side of the Golgi, where budding occurs. Finally, they showed that mice fed a high-fat diet, a model of human metabolic syndrome, increased the levels of PICK1 mRNA in the pituitary, suggesting an involvement of the protein in the development of, or response to, metabolic changes. Similarly, an elevation of PICK1 signal was observed when modeling type 2 diabetes in flies.

Based on these results, both groups propose that, early in the vesicle formation process, PICK1 and ICA69 link together via their BAR domains to form a compound structure essential to the maturation process at the Golgi, possibly through their ability to induce membrane curvature. These results apply at least to vesicles containing either insulin or growth hormone; Holst et al. found some pituitary hormones apparently unaffected by loss of PICK1. It is possible, though still untested, that a subset of human cases of growth hormone deficiency or diabetes may result from loss of one or the other of the two proteins. Regardless, identification of the important role of the pair will likely lead to even further elucidation of the mechanisms through which hormones are packaged and released throughout the body.

**Cao M, Mao Z, Kam C, Xiao N, Cao X, et al. (2013) PICK1 and ICA69 Control Insulin Granule Trafficking and Their Deficiencies Lead to Impaired Glucose Tolerance. doi:10.1371/journal.pbio.1001541**

**Holst B, Madsen KL, Jansen AM, Jin C, Rickhag M, et al. (2013) PICK1-Deficiency Impairs Secretory Vesicle Biogenesis and Leads to Growth Retardation and Decreased Glucose Tolerance. doi:10.1371/journal.pbio.1001542**

